# Subtraction of single-photon emission computed tomography (SPECT) in radioembolization: a comparison of four methods

**DOI:** 10.1186/s40658-024-00675-7

**Published:** 2024-08-15

**Authors:** Camiel E. M. Kerckhaert, Hugo W. A. M. de Jong, Marjolein B. M. Meddens, Rob van Rooij, Maarten L. J. Smits, Yothin Rakvongthai, Martijn M. A. Dietze

**Affiliations:** 1 Radiology and Nuclear Medicine, Utrecht University and University Medical Center Utrecht, P.O. Box 85500, 3508 GA Utrecht, Netherlands; 2 Chulalongkorn University Biomedical Imaging Group, Department of Radiology, Faculty of Medicine, Chulalongkorn University, Bangkok, Thailand; 3 Division of Nuclear Medicine, Department of Radiology, Faculty of Medicine, Chulalongkorn University, Bangkok, Thailand

**Keywords:** SPECT, Subtraction, Iterative image reconstruction, Digital simulation, Phantom study

## Abstract

**Background:**

Subtraction of single-photon emission computed tomography (SPECT) images has a number of clinical applications in e.g. foci localization in ictal/inter-ictal SPECT and defect detection in rest/stress cardiac SPECT. In this work, we investigated the technical performance of SPECT subtraction for the purpose of quantifying the effect of a vasoconstricting drug (angiotensin-II, or AT2) on the Tc-99m-MAA liver distribution in hepatic radioembolization using an innovative interventional hybrid C-arm scanner. Given that subtraction of SPECT images is challenging due to high noise levels and poor resolution, we compared four methods to obtain a difference image in terms of image quality and quantitative accuracy. These methods included (i) image subtraction: subtraction of independently reconstructed SPECT images, (ii) projection subtraction: reconstruction of a SPECT image from subtracted projections, (iii) projection addition: reconstruction by addition of projections as a background term during the iterative reconstruction, and (iv) image addition: simultaneous reconstruction of the difference image and the subtracted image.

**Results:**

Digital simulations (XCAT) and phantom studies (NEMA-IQ and anthropomorphic torso) showed that all four methods were able to generate difference images but their performance on specific metrics varied substantially. Image subtraction had the best quantitative performance (activity recovery coefficient) but had the worst visual quality (contrast-to-noise ratio) due to high noise levels. Projection subtraction showed a slightly better visual quality than image subtraction, but also a slightly worse quantitative accuracy. Projection addition had a substantial bias in its quantitative accuracy which increased with less counts in the projections. Image addition resulted in the best visual image quality but had a quantitative bias when the two images to subtract contained opposing features.

**Conclusion:**

All four investigated methods of SPECT subtraction demonstrated the capacity to generate a feasible difference image from two SPECT images. Image subtraction is recommended when the user is only interested in quantitative values, whereas image addition is recommended when the user requires the best visual image quality. Since quantitative accuracy is most important for the dosimetric investigation of AT2 in radioembolization, we recommend using the image subtraction method for this purpose.

## Introduction

Subtraction of single-photon emission computed tomography (SPECT) images can be performed to visualize and quantify anatomical or functional changes in a patient. SPECT subtraction has a number of long standing clinical applications, including subtraction of inter-ictal and ictal SPECT images, rest and stress cardiac SPECT, and of combined Tc-99m-macro aggregated albumin (MAA) Tc-99m-Sulfur Colloid and Tc-99m-MAA SPECT images for automatic dosimetry in radioembolization [1–3].

In this work, we investigate the performance of SPECT subtraction for a new application: to study the effect of a vasoconstrictor drug during hepatic radioembolization. In a radioembolization procedure, radioactive microspheres are injected in the hepatic artery after which they accumulate in the small (tumor) vessels [4, 5]. A radioembolization treatment is preceded by a safety procedure in which Tc-99m-MAA is injected to mimic the behavior of the microspheres. The Tc-99m-MAA distribution is evaluated for tumor targeting and extrahepatic depositions by quantitative analysis of a SPECT scan.

To achieve the best radioembolization treatment outcome, it is crucial that the healthy liver dose is kept low and the tumor dose is maximized. Previously, intra-arterial injection of a vasoconstricting drug (angiotensin II; AT2) has been suggested to improve the uptake in the tumors by predominantly constricting blood vessels in healthy tissue [6, 7]. In an upcoming clinical trial, we aim to investigate the quantitative effect of AT2 on the distribution of Tc-99m-MAA in the liver.

**Fig. 1:**
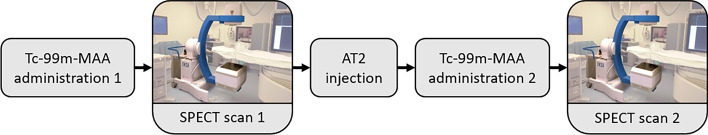
Overview of the clinical trial protocol in which the interventional hybrid scanner (IXSI) developed at the University Medical Center Utrecht is depicted

Normally, a trial of this type would require two procedures executed on separate days: one procedure performed with AT2 and one procedure without, with the acquisition of SPECT/CT at the nuclear medicine department after both procedures. However, in the University Medical Center Utrecht (Utrecht, the Netherlands), we have developed a mobile hybrid c-arm scanner that can perform simultaneous SPECT and cone beam CT scans in the intervention room (named Interventional X-ray and Scintigraphy Imaging, or IXSI), giving a unique opportunity to investigate the impact of AT2 on individual tumors in a single-session procedure [8–10]. Here, half of the Tc-99m-MAA is injected and an in-room SPECT scan is acquired, followed by injection of the second half of Tc-99m-MAA (shortly after AT2 infusion) and the acquisition of another SPECT scan (see Fig. 1).

A single-session procedure is preferred over two separate sessions since there is no disease progression between the two procedures, the catheter can stay in place (as tumor uptake can be sensitive to catheter positioning [11]), and the burden on the patient is minimized since no additional intervention is required. However, it is more challenging from a technical point of view since the second SPECT acquisition will be a combination of the first MAA distribution (without AT2) and the second (AT2 modified) MAA distribution, requiring a SPECT subtraction to retrieve the AT2 modified MAA distribution only [12].

It can be challenging to subtract SPECT images from each other because of their high noise levels. SPECT subtractions are commonly performed using straightforward voxel-wise subtraction of two images but this may not be optimal for the application proposed in this work. Therefore, we additionally evaluated three other methods to obtain the difference image: subtraction of projections, addition of projections during iterative reconstruction, and addition of images within a joint iterative reconstruction. The aim of this work was to compare these four methods against conventional SPECT imaging. Thereby, we investigated whether SPECT images generated by subtraction of scans from a single radioembolization procedure provide sufficient diagnostic information to evaluate the effect of AT2 in a clinical study. Visual quality and quantitative accuracy were evaluated using digital simulations and phantom experiments.

## Methods

**Fig. 2:**
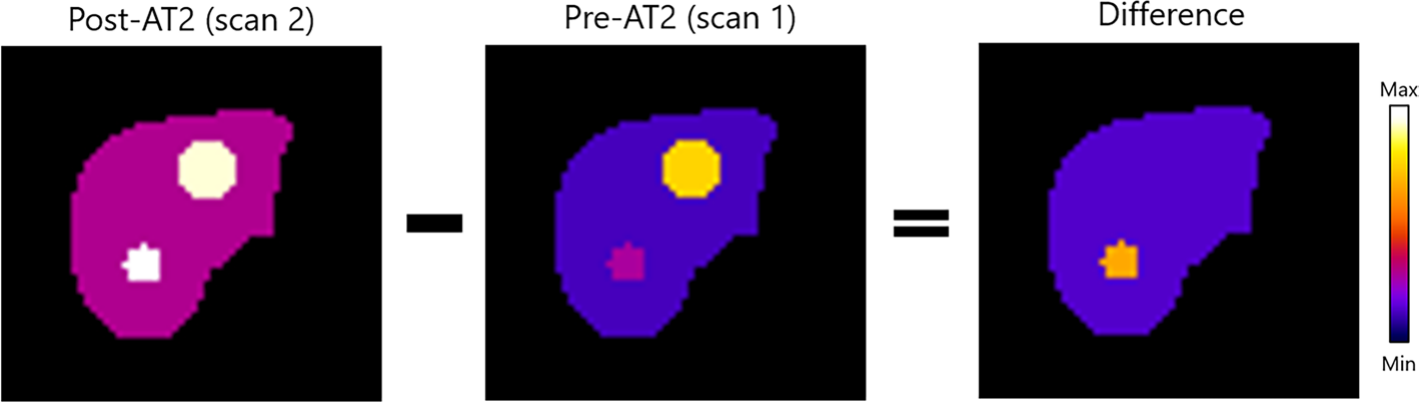
Trans axial slice from activity maps used in the simulation study as post-AT2 (left), pre-AT2 (middle) and difference (right). Two tumors were included in the activity maps: tumor 1 with a diameter of 3 cm (T/N ratio of 2.00 before AT2 and 4.00 after AT2) and tumor 2 with a diameter of 5 cm (T/N ratio of 5.00 before AT2 and 1.00 after AT2)

Four different methods of subtracting SPECT images were investigated in this study. We define the image being subtracted from another image as the *pre-AT2* (in our case the first SPECT), and the image that it is subtracted from as the *post-AT2* (in our case the second SPECT) as can be seen in Fig. 2. The result from the subtraction will be called the *difference*. The following techniques were included.

1. Image Subtraction

**Fig. 3:**
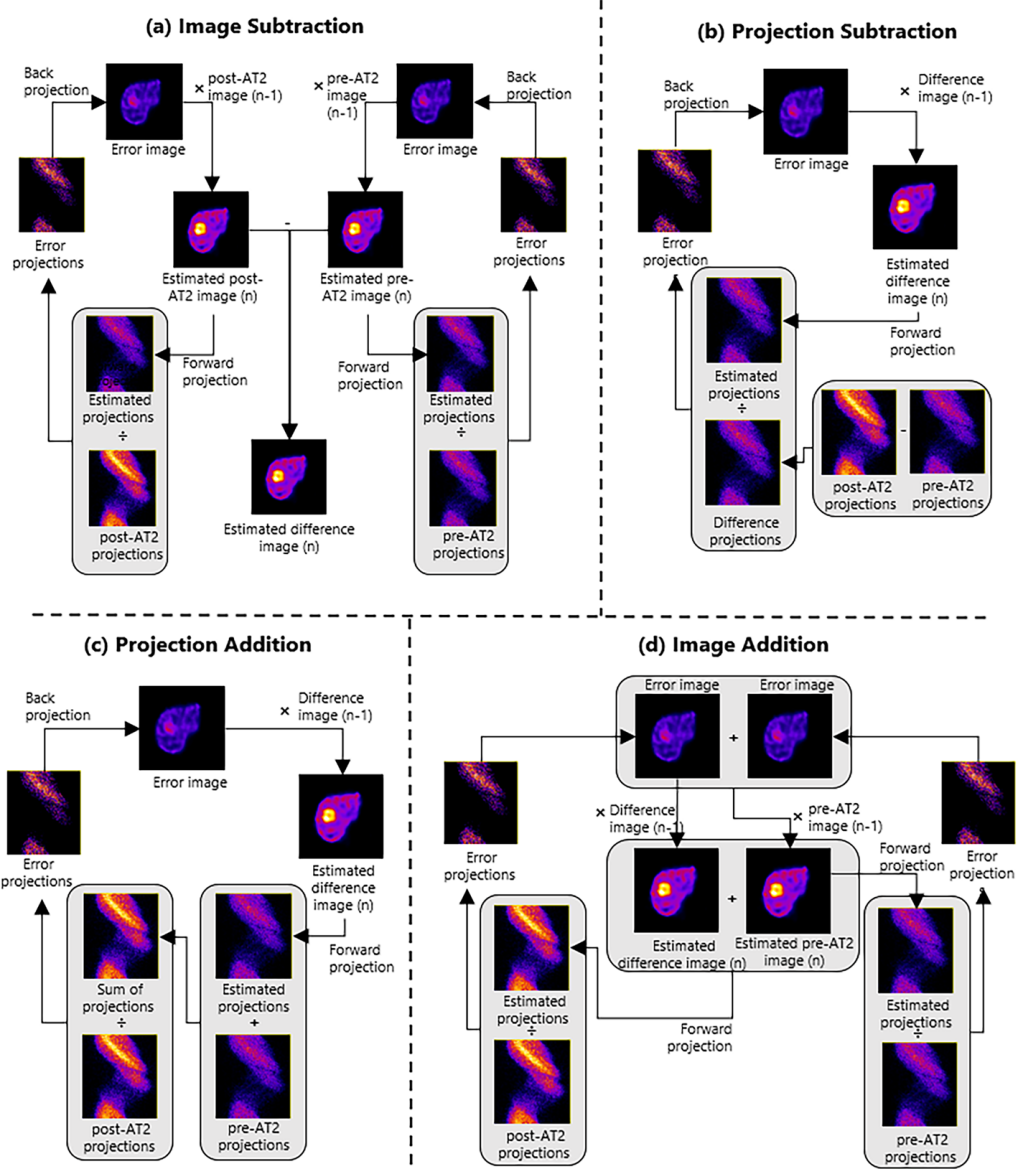
Overview of the subtraction methods evaluated in this study, including image subtraction (**a**), projection subtraction (**b**), projection addition (**c**), and image addition (**d**)

In image subtraction, two SPECT images were reconstructed separately (with the same number of iterations). Subsequently, the pre-AT2 image was subtracted from the post-AT2 image as can be seen in Fig. 3a. Negative values in the difference image were allowed.

(2) Projection Subtraction

In projection subtraction, the measured projections of the pre-AT2 scan were subtracted from the measured projections of the post-AT2 scan (see Fig. 3b). Negative values in the resulting difference projections were set to zero. The difference projections were then used to reconstruct a difference image.

(3) Projection Addition

Subtraction by projection addition was based on a method of scatter compensation during iterative SPECT reconstruction, and was performed by addition of the measured pre-AT2 projections to the estimated difference projections during the iterative reconstruction process [13]. This sum of projections was iteratively corrected using the measured post-AT2 projections (see Fig. 3c).

(4) Image Addition

In image addition, the difference image was created by summing the image estimates of the difference image and the pre-AT2 image, and using the post-AT2 projections for correction. Consequently, these images were simultaneous reconstructed as can be seen in Fig. 3d. This method was based on the joint reconstruction method proposed by Rakvongthai et al. [14]. The joint reconstruction method originally contained interscan misregistration compensation which was not included in the image addition technique.

The reconstructions obtained from the four subtraction methods were compared to the reconstruction quality that would be obtained if the distribution with AT2 was separately acquired and reconstructed (i.e. as in a two procedures protocol), hereafter referred to as the conventional reconstruction.

### Image acquisition

The hybrid C-arm scanner (IXSI) was used to perform the phantom experiments and as a model for the digital simulations [8–10]. The scanner comprises a dual-layer detector and an X-ray tube mounted on a C-arm gantry that can perform non-circular rotation closely around the patient. The dual-layer detector is composed of an x-ray flat panel detector and a gamma camera (equipped with a low-energy high-resolution cone-beam collimator). This design enables simultaneous acquisition of overlapping fluoroscopic and nuclear projections [9]. Scanning was performed in 120 nuclear projections distributed over 360 degrees. The duration of the scans was ten minutes, which is relatively short to minimize the burden on the patient during the intervention.

Images were reconstructed using the Utrecht Monte Carlo System (UMCS) software package, which corrected for photon attenuation, collimator resolution through point spread function modeling, and scatter through fast Monte Carlo simulation. Ordered-subset expectation–maximization (OSEM) reconstruction was used as reconstruction algorithm [15]. Images were reconstructed in iterations of eight subsets to 108 × 108x96 voxels with a size of 4.8 × 4.8x4.8 mm^3^. No post-processing filters were applied to the SPECT reconstructions. A photo peak window between 129.5 and 150.5 keV was applied.

### Simulation study

The digital XCAT phantom was used to generate activity maps and an accompanying attenuation map that resemble realistic patient data with radioactivity in the liver parenchyma and tumors, as can be seen in Fig. 2 [16].

As patients outside of the clinical trial would receive 150 MBq of Tc-99m-MAA, the default phantom configurations (used to model a patient with and without AT2) each contained a total activity of 75 MBq. Phantoms included two liver tumors with diameters of 30 and 50 mm (tumors 1 and 2, respectively) to represent hepatocellular carcinoma (HCC) patients [17]. Based on previous findings, tumors in the configuration without AT2 had tumor-to-nontumor activity ratios (T/N ratios) of 2.00 and 5.00, respectively, and those in the configuration with AT2 4.00 and 1.00, respectively [6]. This phantom configuration simulates a situation in which AT2 doubles the T/N ratio of one tumor, whereas the activity in the other tumor depletes. Beside these default configurations, experiments were performed with varying tumor size (10–80 mm), total activity (50–250 MBq) and activity split (between first and second administration). In each experiment, only one parameter was changed.

UMCS was used to simulate noise-free nuclear projections, after which Poisson noise scaled to the radioactivity level was added to the projections. Ten noise realizations were simulated for each experimental setting.

### Phantom study

Two phantoms were scanned to validate the simulation results with the hybrid C-arm scanner. The NEMA image quality (IQ) phantom and anthropomorphic torso phantom were scanned three times: in the pre-AT2, post-AT2, and difference configurations. Every scan was repeated five times to assess the consistency of various measurements.

The NEMA IQ phantom contained 6 spheres of 10, 13, 17, 22, 28 and 37 mm in diameter in a circular pattern. All spheres had T/N ratios of 4.00, 6.00 and 8.00 with total phantom activities of 150, 300, and 150 MBq for the pre-AT2, post-AT2 and difference configurations, respectively.

The anthropomorphic phantom included a liver volume of 1172 ml in which one sphere of 15.9 ml and a hollow sphere with a volume of 18.9 ml were placed. Both spheres had T/N ratios of 2.00, 3.00 and 4.00 with total phantom activities of 75, 150 and 75 MBq for the pre-AT2, post-AT2 and difference configurations, respectively.

The following steps were taken to obtain an attenuation map to include in the SPECT reconstruction: The X-ray projections that were acquired simultaneously with the SPECT acquisition were reconstructed into a cone beam CT (CBCT) volume using the ASTRA toolbox with a SIRT algorithm [18, 19]. An attenuation map that was previously acquired on a clinical SPECT/CT systems (Siemens Symbia) was then translationally registered to the CBCT reconstruction using the Elastix registration tool [20]. In the proposed AT2 investigation, this attenuation map would be derived from a previously acquired CT scan, which is typically available for radioembolization patients.

### Evaluation

The quality of the reconstructed images was assessed visually and quantitatively. Metrics were calculated over volumes of interest (VOI) using tumor masks and a background mask (representing the healthy liver tissue). In the simulation study, masks were created using a threshold on the phantom activity maps. In the phantom study, masks were manually created over the registered CT images. For the background masks, a 3D erosion of three pixels was applied to mitigate partial volume effects. Paired t-tests were performed to determine whether differences between subtraction techniques were significant. The following metrics were included for quantitative evaluation:

*Activity Recovery Coefficient* (ARC), as a measure of the accuracy of the reconstructed activity

1 $$ARC= \frac{\langle {a}_{estimate}\rangle }{\langle {a}_{true}\rangle }*100\text{\%}$$

where $\langle {a}_{estimate}\rangle$ and $\langle {a}_{true}\rangle$ are the mean measured activity in the reconstruction or subtraction image and the true mean activity, respectively.

*Noise*, measured as normalized standard deviation over a uniform background of N pixels.

2 $$\text{Noise}= \frac{1}{\langle {\text{a}}_{\text{BG}}\rangle }\sqrt{\frac{{\sum_{\text{n}}^{\text{N}}({\text{a}}_{\text{BG}}\left(\text{n}\right)-\langle {\text{a}}_{\text{BG}}\rangle )}^{2}}{\text{N}-1}}$$

where $\langle {a}_{BG}\rangle$ is the mean measured activity in the background of the reconstruction or subtraction image.

*Contrast-to-noise ratio* (CNR), calculated from the contrast between tumor and background, and the noise (Eq. 2).

3 $$\text{Contrast}= \frac{\langle {\text{a}}_{\text{tumor}}\rangle - \langle {\text{a}}_{\text{BG}}\rangle }{\langle {\text{a}}_{\text{BG}}\rangle }$$

4 $$\text{CNR}= \frac{\text{Contrast}}{\textrm{Noise}}$$

*Tumor-to-nontumor ratio (T/N ratio)*, calculated from the mean activity in a tumor, and the mean activity in the background.

5 $$T/N ratio= \frac{\langle {a}_{tumor}\rangle }{\langle {a}_{BG}\rangle }$$

As dosimetry is our main focus, the optimal iteration number per method was defined as the first iteration in which the increase in ARC of both the tumors and the background has become lower than 1%. To this end, 25 iterations were first performed for each method.

## Results

### Digital simulations

**Fig. 4:**
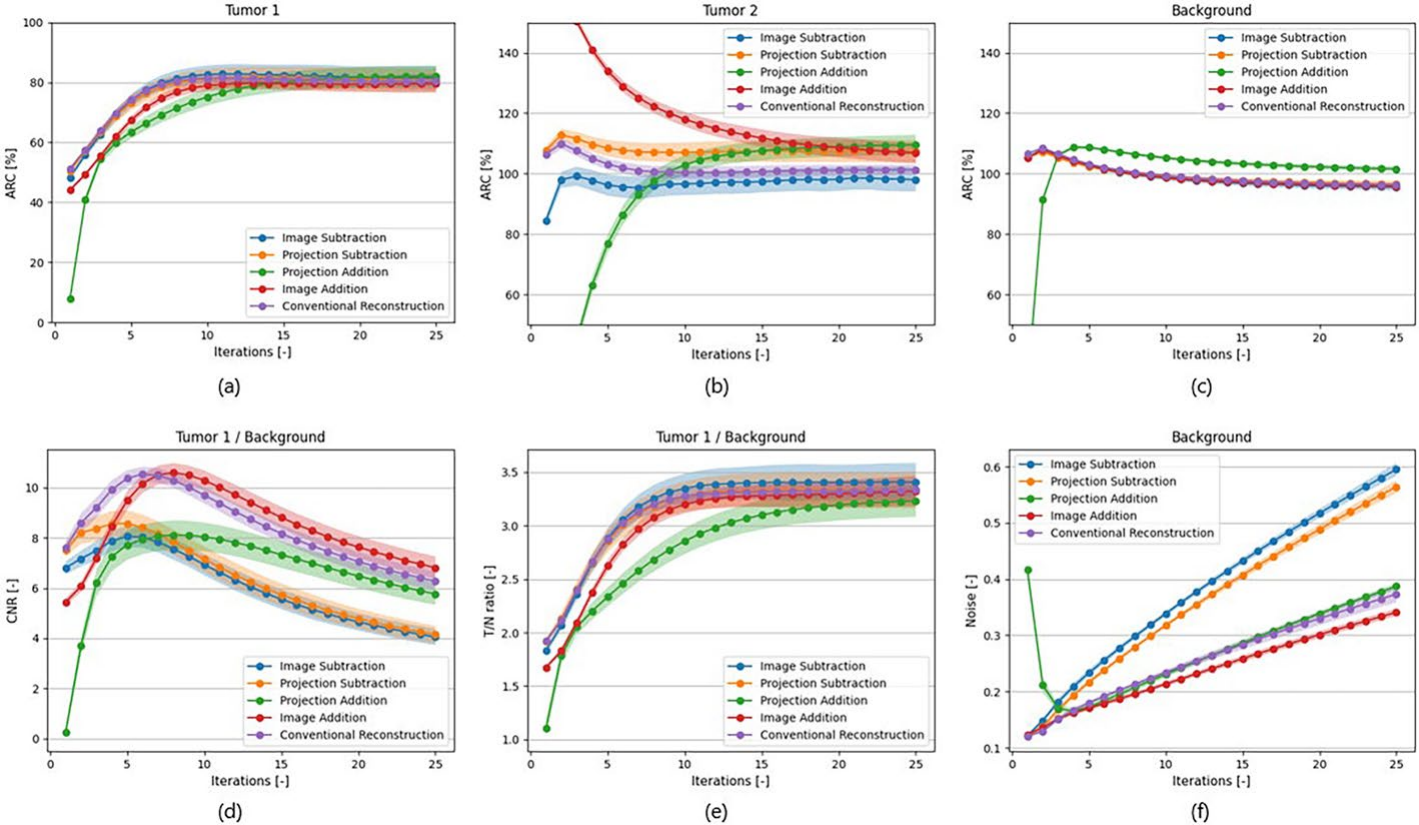
ARC (**a**, **b**, **c**), CNR (**d**), T/N ratios (**e**), and Noise levels (**f**) of Tumor 1 (true T/N ratio of 4.00), Tumor 2 (true T/N ratio of 1.00) and background resulting from the subtraction methods and conventional reconstruction at increasing numbers of reconstruction iterations. The lines and error bars indicate the mean and standard deviation over 10 noise realizations

Figure 4 shows ARC, CNR, T/N ratio and noise levels at each iteration for the default phantoms. The ARC of tumor 2 (Fig. 4b) shows that projection addition and image addition require more iterations before stabilization. Furthermore, the background ARC (Fig. 4c) shows that projection addition consistently overestimates the mean intensity of the background.

**Fig. 5:**
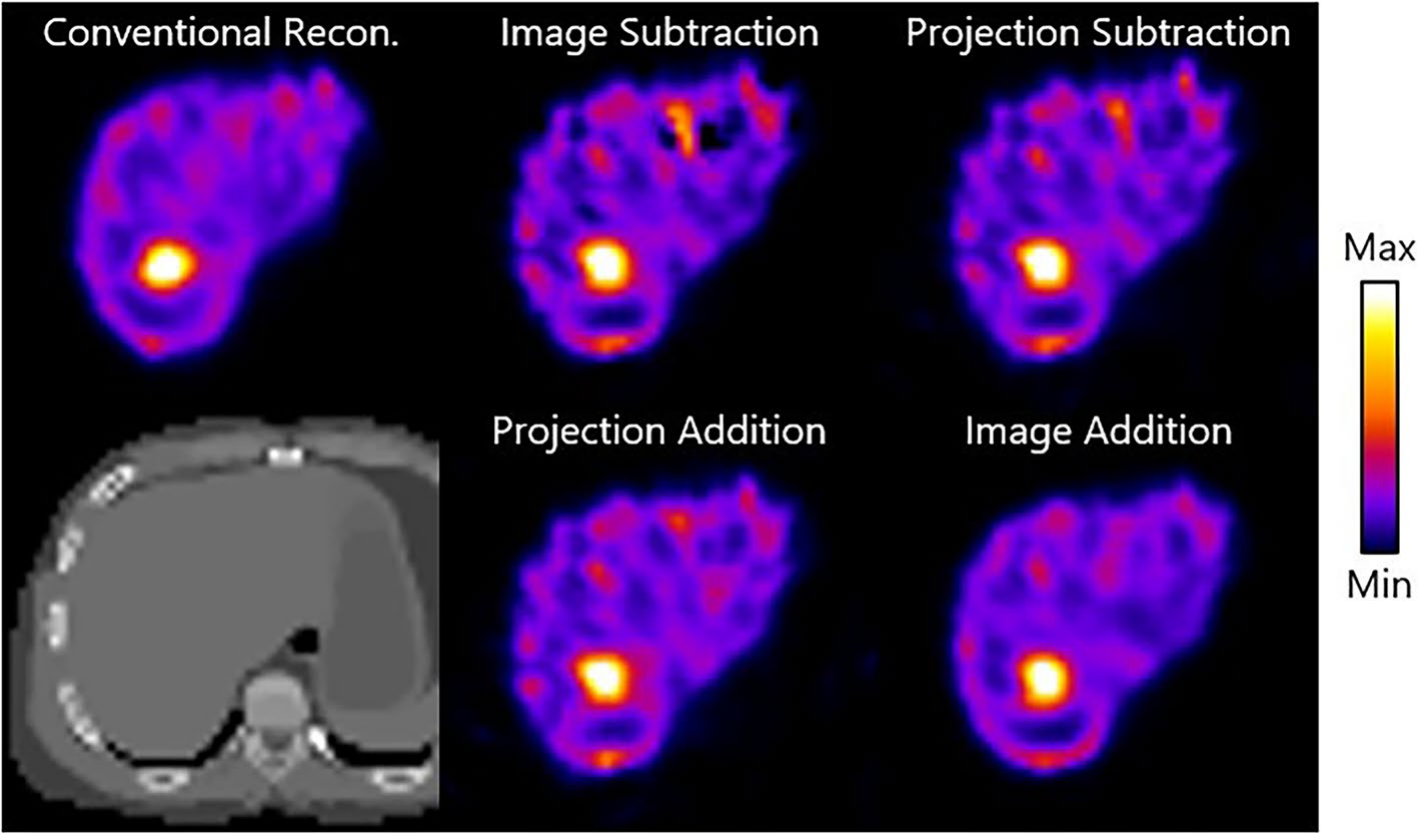
Example results from the simulation study: trans axial slices of difference images from the XCAT phantom using conventional reconstruction, image subtraction, projection subtraction, projection addition and image addition, after the optimal number of iterations. A corresponding CT view (bottom left) is shown as well. The phantoms shown have a true total activity of 75 MBq and the visible tumor has a true T/N ratio of 4.00

Representative reconstructed images at the optimal number of iterations are shown in Fig. 5. The difference image from the image addition method is visually the most similar to the conventional reconstruction in terms of sharpness and noise level. The shape of tumor 2, which should not be visible due to its T/N ratio of 1.00 in the difference image, can be seen in the difference image from image subtraction.

Metric values at the respective optimal iterations of each method have been listed in Table 1. The CNR should be as high as possible, and noise as low as possible. T/N ratio (ideally 4.00) and ARC (ideally 100%) should be as close to the phantom configuration as possible. Image addition significantly outperformed the other methods in terms of noise and CNR (*p* values < 0.01), while image subtraction was superior in terms of T/N ratio and ARC in the tumors (*p* values ≤ 0.01).

**Table 1:** Mean and standard deviation of the CNR, Noise, T/N ratio, and ARC for each of the subtraction methods and conventional reconstruction of the XCAT phantom in the simulation study

	Optimal number of iterations​	CNR [-]	Noise [-]	T/N ratio [-]	ARC [%]		
		*Tumor 1 Background*	*Background*	*Tumor 1 Background​*	*Tumor 1*	*Tumor 2*	*Background*
Conventional reconstruction	8	10.30 ± 0.30​	0.21 ± 0.00​	3.20 ± 0.07	80.41 ± 1.61​	100.61 ± 2.03​	100.48 ± 0.42​
Image subtraction	9	7.26 ± 0.41​	0.32 ± 0.00​	**3.32 ± 0.13**	**82.13 ± 2.85​**	**96.48 ± 3.77​**	99.11 ± 0.81​
Projection subtraction	9	7.49 ± 0.50​	0.30 ± 0.00​	3.24 ± 0.14	80.67 ± 3.13​	107.06 ± 3.19​	**99.63 ± 0.70​**
Projection addition	13	7.67 ± 0.54​	0.26 ± 0.01​	3.03 ± 0.13	78.72 ± 3.13​	106.50 ± 3.14​	103.92 ± 0.72​
Image addition	12	**9.73 ± 0.41​**	**0.23 ± 0.00​**	3.25 ± 0.09	79.60 ± 1.94​	114.96 ± 2.65​	97.83 ± 0.57​

Metrics are calculated at the optimal number of iterations shown in the table. The best performing subtraction method per measurement is depicted in bold

### Activity split, total activity and tumor size

**Fig. 6:**
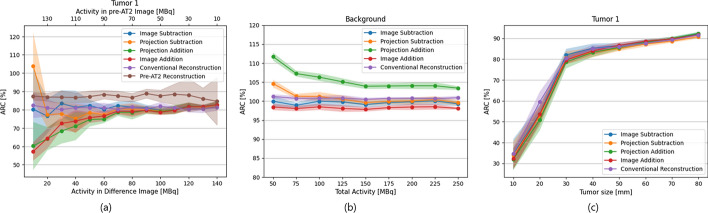
ARC of Tumor 1 or background for the subtraction methods, the conventional difference image reconstruction (and conventional pre-AT2 image reconstruction) for varying activity splits between pre-AT2 and difference image (**a**), total activities (**b**) and tumor sizes (**c**). The lines and error bars indicate the mean and standard deviation over 10 noise realizations

In Fig. 6a, the ARC values of tumor 1 are shown at varying activity splits. Here, activity split refers to the distribution of 150 MBq (the normal amount of activity used in the hepatic radioembolization safety procedure) between the first injection (pre-AT2 image) and the second injection (difference image). As the activity split affects the pre-AT2 image quality, metrics calculated on that image are shown in Fig. 6a as well. The figure shows that image subtraction is the most robust method over all activity splits and is thereby most similar to conventional reconstruction. From the figure becomes clear that both the pre-AT2 image and difference images show higher standard deviations in ARC as their respective total activity decreases. The subtraction methods are found to perform well (i.e. no bias and low standard.

deviation) between 60 and 90 MBq in the pre-AT2 image (pre-vasoconstrictor injection). Hence, a 75 MBq/75 MBq combination as was previously suggested would be optimal for a clinical study.

Additionally, Fig. 6b shows the ARC of the background at different total activities over the two Tc-99m-MAA injections, ranging from 50 to 250 MBq. Noticeably, projection subtraction and projection addition show an increase in ARC as the total activity decreases, while the other methods remain at a similar ARC level.

To investigate variance in patients and disease stage, different tumor sizes were simulated. To determine the smallest tumor volume that would render a feasible difference reconstruction, tumors varying in size from 10 to 80 mm in diameter were simulated. Figure 6c depicts the resulting ARC values. Especially tumors with a diameter of less than 30 mm resulted in substantially lower ARCs than larger tumors, but no substantial differences with the conventional reconstruction are found. This indicates that tumor size does not need to be a restricting factor for choosing either a multiple-day or single-session procedure.

### Phantom study

**Fig. 7:**
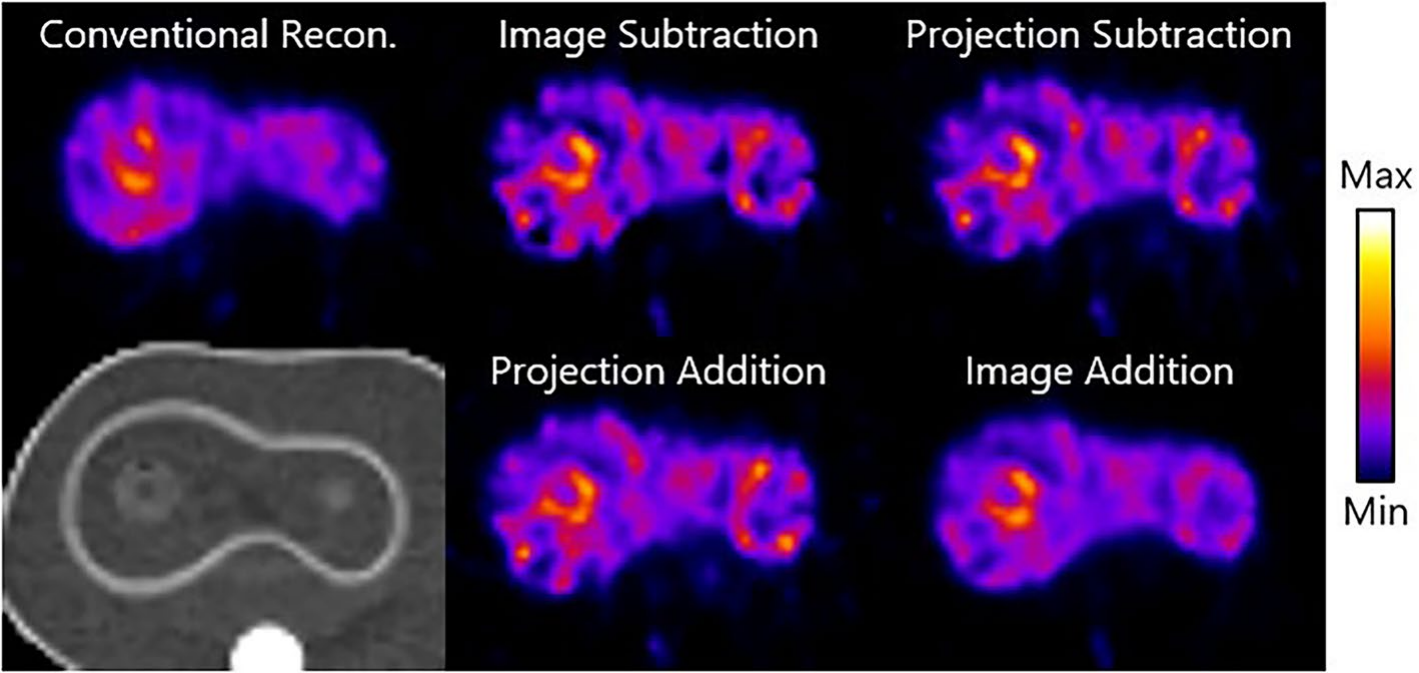
Exemplary images from the phantom study: trans axial slices of difference images from the anthropomorphic torso phantom using conventional reconstruction, image subtraction, projection subtraction, projection addition, and image addition after the optimal number of iterations. A corresponding CT view (bottom left) is shown as well. The phantoms shown have a true total activity of 75 MBq and the tumors have a true T/N ratio of 4.00

Figure 7 shows representative images from conventional reconstruction and the subtraction methods of the anthropomorphic torso phantom. The optimal number of iterations for each method found in the simulation study was used in the phantom study as well. Comparable to the simulation study, the lowest noise level in the anthropomorphic torso phantom images was achieved by image addition. In the depicted trans axial slices, the hollow sphere is visible and appears to be similarly sharp for all subtraction methods.

The noise levels, CNR and ARC values of three VOIs of the anthropomorphic phantom calculated at the optimal number of iterations are shown in Table 2. Similarly to the simulation study the image addition method is superior in terms of noise and CNR, whereas none of the methods is superior in terms of ARC and T/N ratio.

**Table 2:** Mean and standard deviation of the CNR, Noise, T/N ratio, and ARC for each of the subtraction methods and conventional reconstruction in the anthropomorphic phantom in the phantom study

	CNR (-)		Noise (-)	T/N ratio (-)	ARC (%)		
	Normal sphere Background	Hollow sphere Background	Background	Normal sphere Background	Normal sphere	Hollow sphere	Background
Conventional reconstruction​	8.49 ± 0.83	2.71 ± 0.47	0.32 ± 0.05	3.69 + -0.16	85.36 ± 5.93	42.76 ± 1.44	92.54 ± 3.9
Image subtraction​	4.50 ± 0.29	2.36 ± 0.41	0.50 ± 0.05	3.23 + -0.19	67.08 ± 3.26	**44.84 ± 3.46**	84.37 ± 2.28
Projection subtraction​	4.88 ± 0.36	2.63 ± 0.48	0.42 ± 0.04	3.05 + -0.14	63.33 ± 2.33	43.56 ± 3.48	84.26 ± 2.23
Projection addition​	5.44 ± 0.39	2.54 ± 0.45	0.39 ± 0.03	3.11 + -0.09	**68.42 ± 0.94**	43.57 ± 3.41	**89.45 ± 2.01**
Image addition​	**6.22 ± 0.81**	**3.27 ± 0.76**	**0.37 ± 0.08**	**3.26 + -0.17**	66.47 ± 2.93	44.18 ± 3.08	82.78 ± 1.91

Metrics are calculated per VOI at the optimal number of iterations. The best performing subtraction method per measurement is depicted in bold

**Fig. 8:**
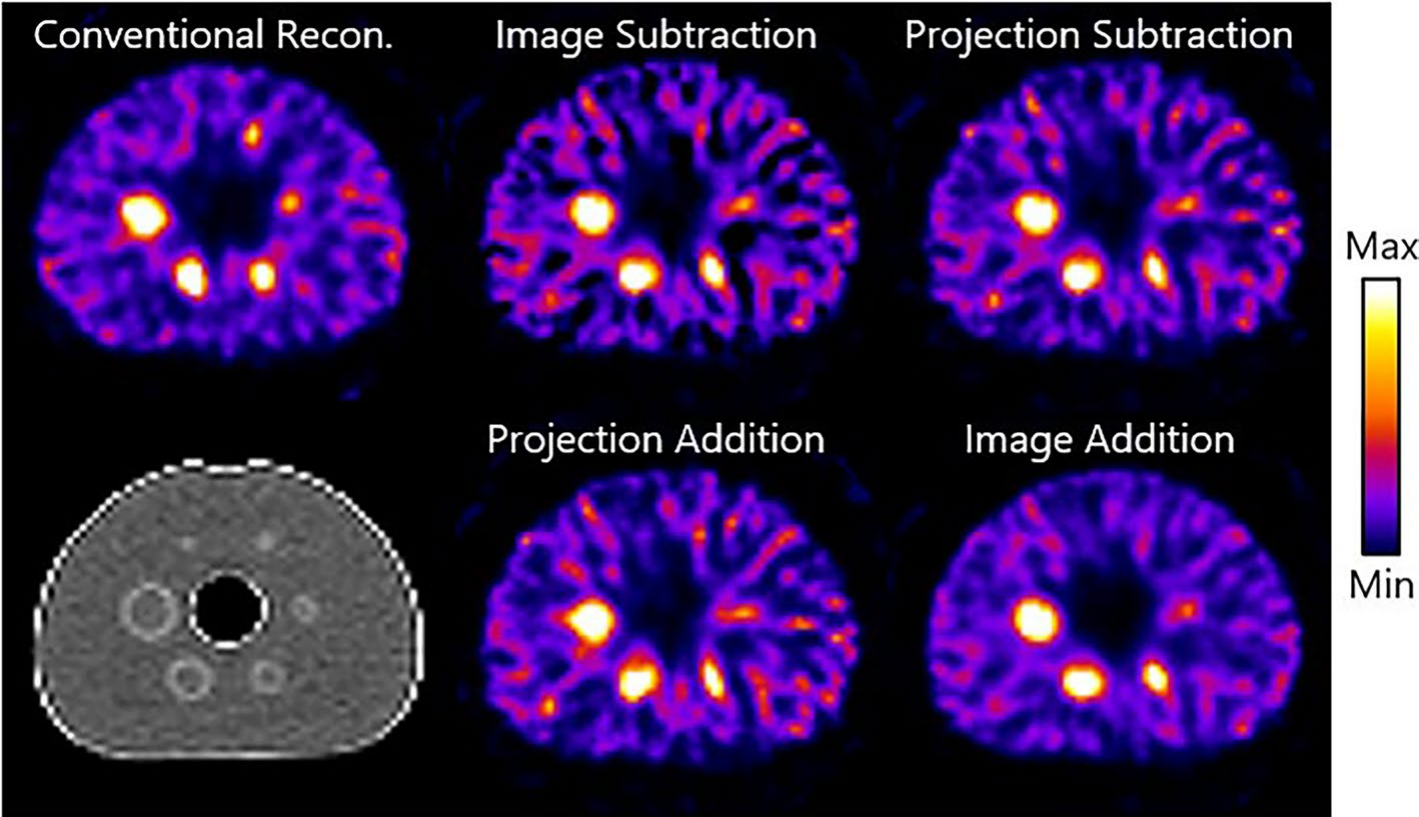
Exemplary images from the phantom study: trans axial slices of difference images from the NEMA phantom using conventional reconstruction, image subtraction, projection subtraction, projection addition, and image addition after the optimal number of iterations. A corresponding CT view (bottom left) is shown as well. The phantoms shown have a true total activity of 150 MBq and the tumors have a true T/N ratio of 8.00.

Figure 8 shows a conventionally reconstructed image and difference images from the subtraction techniques of the NEMA phantom. Again, the image addition resulted in the superior image quality in terms of noise which is comparable to the conventional method. Noticeable, however, is that the smaller (≤ 17 mm in diameter) spheres are less sharp and visible in the difference images compared to the conventional reconstruction.

Table 3 depicts the ARC values of the subtraction methods and a conventional reconstruction calculated for the different sphere sizes. Even though high standard deviations cause overlap between method performances, image subtraction shows to be superior in terms of activity recovery in five out of the seven ROIs.

**Table 3:** Mean and standard deviation of the noise and ARC for each of the subtraction methods and conventional reconstruction in the NEMA phantom in the phantom study

	Noise (-)				ARC (%)			
	Background	37 mm	28 mm	22 mm	17 mm	13 mm	10 mm	Background
Conventional reconstruction	0.37 ± 0.01	58.41 ± 3.19	52.50 ± 3.73	37.73 ± 3.88	29.86 ± 1.69	19.30 ± 2.79	14.77 ± 4.29	87.24 ± 3.54
Image subtraction	0.64 ± 0.11	**63.49 ± 9.93**	**54.43 ± 10.13**	**43.17 ± 6.06**	**23.57 ± 7.23**	**21.96 ± 7.35**	12.33 ± 4.06	94.83 ± 23.75
Projection subtraction	0.60 ± 0.13	57.55 ± 6.44	47.19 ± 10.87	38.12 ± 8.91	21.14 ± 5.66	20.09 ± 7.46	11.46 ± 2.67	93.33 ± 17.83
Projection addition	0.63 ± 0.21	59.32 ± 6.95	46.02 ± 15.70	36.35 ± 13.85	21.57 ± 5.77	21.74 ± 8.13	12.48 ± 2.58	**97.92 ± 19.00**
Image addition	**0.48 ± 0.12**	62.88 ± 9.93	53.08 ± 10.58	40.93 ± 6.36	21.39 ± 7.25	20.99 ± 7.36	**12.90 ± 3.49**	93.59 ± 21.68

Metrics are calculated per VOI, for which the sphere diameter sizes as mentioned in the table. Metrics are calculated at the optimal number of iterations. The best performing subtraction method per measurement is depicted in bold

## Discussion

This study evaluated the quality of four SPECT subtraction techniques and compared their results to a separate acquisition with conventional reconstruction. Qualitatively sufficient SPECT subtraction would enable the investigation of the effect of AT2 in radioembolization by employing a single treatment session with two activity administrations and scans made with a hybrid c-arm scanner. While our findings are relevant for many different imaging scenarios (as both positive and negative effects were simulated), we had a focus on the specific case of this AT2 study.

Difference images obtained with image subtraction had the highest noise level. Nevertheless, this method demonstrated the best quantitative accuracy out of all subtraction methods. To investigate robustness against spatial changes, the simulation study incorporated a tumor that was present before AT2 injection (T/N ratio of 5.00) but disappeared after AT2 injection (T/N ratio of 1.00). For image subtraction, this resulted in a visual anomaly but the quantitative performance was not noticeably affected.

Projection subtraction performed similarly to image subtraction by showing generally accurate activity recoveries and noisy images. However, this method resulted in an overestimated ARC for the depleting tumor. Presumably, this overestimation originates from the removal of negative values in the subtracted projections, which have to be removed to perform the back projections during reconstruction. Still, the depleting tumor resulted in less visible effects compared to the image subtraction method.

Projection addition proved to be able to improve the visual quality of the difference image as it yielded fewer noise compared to image and projection subtraction. However, this method resulted in consistent quantitative inaccuracies as ARC values were increasingly overestimated as the total activity decreased. This increase can also be seen in projection subtraction and therefore presumably also stems from the removal of negative projection values before back projection.

Finally, image addition yielded generally good quantitative results and a superior image quality comparable to conventional reconstruction, which is aligned with findings from the study that originally proposed this method [14]. However, like projection subtraction and projection addition, this method proved sensitive to differences in anatomical features between pre-AT2 and difference images, as it resulted in overestimated ARC for the depleting tumor. Presumably, this stems from the difference image incorporating too much information from the post-AT2 image (which is used for correction of the estimated projections in the algorithm).

SPECT subtraction requires exact alignment of the pre-AT2 and post-AT2 images. This is ensured in this study by positioning the phantoms in the same way throughout the acquisition of all scans. However, in a clinical context involving unanesthetized patients, achieving this alignment would be more challenging and hence image registration may be necessary. For the subtraction methods that operate in the projection domain (projection subtraction and projection addition), registration would not be straightforward and requires additional research to determine its feasibility. In contrast, the methods that operate in the image domain (image subtraction and image addition) have several opportunities for image registration. For the proposed clinical study, we expect that changes in patient positioning will be small because the patients stay on the table and the time between SPECT scans should be kept as low as possible. This combined with a relatively large pixel size (4.8 × 4.8x4.8 mm^3^) presumably minimizes the positioning error. Hence, image registration will presumably not constrain the choice of subtraction method.

An alternative method to examine the effect of AT2 could be VOI-based instead of pixel-based subtraction. Specifically, the mean activities per VOI could be calculated from scans 1 and 2, and subsequently a difference T/N ratio could be derived. This approach offers the advantage of further minimizing the impact of inter-scan patient motion, as VOIs can be independently delineated in each image. However, this method does not produce a difference image, which remains a critical requirement for physicians in many (other) clinical situations. Consequently, this method was not included in this study.

The results of this work indicated that all the subtraction methods yield reasonable SPECT images with distinguishable features, as they all meet the Rose criterion with CNR values above 5 [21]. However, only image subtraction consistently performs similar to conventional reconstruction in terms of quantitative accuracy, and only image addition consistently aligns with conventional reconstruction in terms of image quality. Since for the investigation of AT2 in radioembolization quantitative accuracy is of highest importance (especially T/N ratio), we recommend image subtraction for this purpose.

Besides our radioembolization scenario, SPECT subtraction has a number of other applications with other imaging tasks, which may influence the optimal subtraction method. For instance, in ictal/inter-ictal SPECT subtraction the task is to find a hot spot in the difference image. Such a detection task is mainly helped by good image quality, and image quantification is of lesser importance. Here, image addition would hence be the method of choice.

This study solely made use of the interventional hybrid scanning device developed at the University Medical Centre Utrecht, IXSI, as its application to a single-session radioembolization procedure would be less burdening to the patient. This can be regarded as a worst case scenario in terms of noise level, as the scanner only contains one gamma camera and the intervention acquisition protocol is limited to ten minutes. When a diagnostic dual headed SPECT scanner is used, noise levels will be lower and overall image quality will be better making subtraction more feasible [9]. In that case, the advantage of image addition in visual quality may be smaller.

In this study we propose to investigate the effect of AT-2 using half of the activity typically used in standard procedures, with the remaining half serving as control. Therefore, a limitation of this study is the assumption that the Tc-99m-MAA distribution, and hence the effect of AT-II, is consistent whether 75 or 150 MBq is injected. Previous studies have indicated that holmium-166 and yttrium-90 distributions may vary in selected cases during injection in radioembolization therapy [22, 23]. However, to our knowledge, this has not been demonstrated for Tc-99m-MAA. Furthermore, based on the effects found for holmium-166 and an AT2 study by van de Hoven et al., the effects of AT2 are expected to be substantially larger than variations in biodistribution [6].

Another limitation to this study is the difference in the amount of noise realizations between the digital simulation study and the phantom study. In the phantom study, only five noise realizations were incorporated due to relatively fast activity decay of Tc-99m combined with a scan duration of ten minutes. Ten noise realizations were applied in the digital simulation study to capture more inter-scan variability. Nevertheless, results from the digital simulations and experiments were in agreement with each other.

Many filtering and smoothing techniques have been developed to remove noise from SPECT images, which may be applied to the subtraction methods studied in this work as well [24]. However, as the optimal type and order of filtering will differ per method, such post-processing methods would complicate the comparisons and have therefore not been applied in this study. Another way to compensate for loss in image quality presumably could be increasing the total amount of activity. However, as was shown in Fig. 6, this would cause a higher radiation dose to the patient without improving the quantitative accuracy.

## Conclusion

All four investigated methods of SPECT subtraction demonstrated the capacity to generate a feasible difference between SPECT images. The optimal SPECT subtraction technique depends on the imaging purpose. Image addition proved to be a superior technique for qualitative and visual purposes (e.g. tumor detection), whereas image subtraction was superior for quantitative purposes (e.g. dosimetry). Since quantitative accuracy is of highest importance for the investigation of AT2 in radioembolization, we recommend to use the image subtraction method for this purpose.

## Data Availability

The authors had full control over the data and the information submitted for publication. Data is stored at the University Medical Centre Utrecht, Utrecht, the Netherlands.

## Abbreviations

SPECT: Single photon emission computed tomography
Tc-99m: Technetium-99m
MAA: Macro-aggregated albumin
AT2: Angiotensin-2
CT: Computed tomography
CBCT: Cone-beam computed tomography
IXSI: Interventional x-ray and scintigraphy imaging
UMCS: Utrecht Monte Carlo system
OSEM: Ordered-subset expectation maximization
HCC: Hepatocellular carcinoma
T/N ratio: Tumor-to-nontumor ratio
SIRT: Simultaneous iterative reconstruction technique
VOI: Volume of interest
ARC: Activity recovery coefficient
CNR: Contrast-to-noise ratio
